# Clinical Features, Biochemical Profile, and Response to Standard Treatment in Lean, Normal-Weight, and Overweight/Obese Indian Type 2 Diabetes Patients

**DOI:** 10.1900/RDS.2021.17.68

**Published:** 2021-10-31

**Authors:** Ahmad Faraz, Hamid Ashraf, Jamal Ahmad

**Affiliations:** 1 Assistant Professor, Department of Physiology, Jawahar Lal Nehru Medical College, Aligarh Muslim University, Aligarh, India,; 2 Assistant Professor, Rajiv Gandhi Centre for Diabetes and Endocrinology, Jawahar Lal Nehru Medical College, Aligarh Muslim University, Aligarh, India,; 3 Former Professor of Endocrinology & Dean Faculty of Medicine, Ex-Director, Rajiv Gandhi Centre for Diabetes & Endocrinology, Aligarh Muslim University, Aligarh Diabetes & Endocrinology Super-Speciality Centre, Aligarh, India.

**Keywords:** type 2 diabetes, glycemic control, lean, normal weight, overweight, obese

## Abstract

**BACKGROUND:**

Much evidence is available on the relationship between type 2 diabetes mellitus (T2D) and obesity, but less on T2D in lean individuals.

**AIM:**

This study was conducted in 12,069 T2D patients from northern India to find out which clinical and biochemical features are related to lean, normal weight, and overweight/obese T2D patients.

**METHODS:**

The study was conducted at two endocrine clinics in northern India as a retrospective cross-sectional study. The records of all patients who attended these clinics from January 2018 to December 2019 were screened. After screening 13,400 patients, 12,069 were labelled as type 2 diabetes mellitus according to the criteria of the American Diabetes Association, 2020, and were included in the study. The patients were subdivided into the three groups by their body mass index (BMI): lean (BMI < 18), normal weight (BMI = 18-22.9), overweight/obese (BMI ≥ 23). The study evaluated how the three subgroups responded to standard diabetes management, including antidiabetic medication and lifestyle interventions.

**RESULTS:**

Of a total of 12,069 patients 327 (2.7%) were lean, 1,841 (15.2%) of normal weight, and 9,906 (82.1%) overweight/obese. Lean patients were younger, but had more severe episodes of hyperglycemia. All three subgroups experienced significant improvements in glycemic control during follow-up; HbA1c values were significantly lowered in the overweight/obese group during follow-up compared with baseline.

**CONCLUSIONS:**

While overweight/obese patients could benefit from the improvements in glycemic control achieved by lowering HbA1c, lean and normal-weight patients had more severe and difficult-to-control hyperglycemia.

## 1 Introduction

According to the International Diabetes Federation (IDF), there are currently 77 million people with diabetes living in India, with an increasing trend [1]. Likewise, the prevalence of overweight/obesity in the adult Indian population has doubled (9.0% in 1990 to 20.4% in 2016) [2]. The risk of diabetes in overweight and obese individuals in India is also significantly higher [3]. For many decades obesity has been an important risk factor for the development of diabetes. Nevertheless, apart from the usual obesity-associated type 2 diabetes (T2D) patient stereotype, and additional well-defined diabetes types, there is also a significant number of people with T2D who are either lean or of normal weight. The prevalence of T2D in lean individuals in India has been reported to vary from 1.6% to 26% [4, 5]. The clinical and biochemical profiles of these patients differ from those of classic T2D patients [5]. They have more profound hyperglycemia, and the incidence of microvascular complications such as diabetic neuropathy, retinopathy, and nephropathy is also higher [5]. Only a few studies have revealed a lower incidence of hypertension, dyslipidemia, and coronary artery disease in the lean type 2 diabetics [5, 6].

The subset of T2D patients with low or normal BMI has not been very well categorized. The key pathology appears to be deficient insulin secretion instead of insulin resistance, which is present in the classical obesity-related diabetes type [7]. Several reports have demonstrated a link between lean T2D, poor nutrition, and poverty during the early phase of life. Though there is no definitive evidence from human studies, animal models have demonstrated that protein deficiency in early life can cause a decline in beta-cell mass leading to deficient insulin secretion [8, 9]. The profiles of these patients also differ from latent autoimmune diabetes of adults (LADA) as the autoimmune markers of LADA are not present in the majority of lean T2D patients [5, 10].

In view of this evidence we conducted a clinical study to characterize the clinical presentation, biochemical characteristics, and response to standard treatment of lean, normal-weight, and overweight/obese T2D patients living in northern India.

## 2 Material and methods

### 2.1 Data retrieval and analysis

We conducted a retrospective cross-sectional study in T2D patients who were attending two endocrine centers in northern India. One of the clinics was the Rajiv Gandhi Centre for Diabetes and Endocrinology, J. N Medical College and Hospital, Aligarh Muslim University, Aligarh (Uttar Pradesh), the other was the Diabetes and Endocrinology Super-Speciality Centre, Aligarh (Uttar Pradesh). The records of all patients who attended these clinics from January 2018 to December 2019 were screened. After screening 13,400 patients, 12,069 patients were identified as T2D patients according to ADA criteria, 2020, and were included in the study [11]. Exclusion criteria were:

- Diagnosis of diabetes before age 20 years
- Diagnosis of diabetes mellitus other than T2D, including type 1 diabetes (T1D), gestational diabetes, fibrocalculous pancreatic diabetes, and drug-induced diabetes.

Individuals diagnosed before the age of 20 were not included in the study to reduce the risk of including T1D patients. The study was designed to analyze the differences in clinical presentation, complication profile, and response to standard treatment between lean, normal weight, and overweight/obese T2D patients. The treatment included standard medical treatment of T2D patients including antidiabetic medication. More than half of the patients (62.2%) received two or three oral antidiabetic drugs. Metformin was the most commonly prescribed drug (>90%) followed by sulfonylureas (55.7%). 91% in the lean group and 94% in both the normal-weight and in the overweight/obese group received metformin.

The relevant information regarding demographic and clinical parameters was obtained from the patients’ records, including age, sex, income, area of residence, duration of disease, treatment (including insulin), blood sugar level (fasting and postprandial), HbA1c level, and comorbidities (hypertension, dyslipidemia, diabetic neuropathy, diabetic kidney disease, diabetic retinopathy, and coronary artery disease). Anthropometric data, including height and weight, were used as per record. Body mass index (BMI) was calculated as weight in kg divided by height in m^2^. Individuals were categorized according to their BMI values as “lean” (<18.0 kg/m^2^), “normal” (18.0-22.9 kg/m^2^), “overweight” (23.0-24.9 kg/ m2), and “obese” (>25 kg/m^2^) [12].

The diagnoses of diabetic retinopathy and diabetic neuropathy were made on the basis of historical and clinical examination (vibration perception and 10-g monofilament, ankle jerk, pinprick, temperature sensation). Additionally, all diabetes patients attending our clinic underwent a detailed fundus examination after dilation by a trained ophthalmologist for confirming diabetic retinopathy diagnosis. Diabetic kidney disease was diagnosed by the presence of albuminuria and/or reduced estimated glomerular filtration rate (according to Cockcroft-Gault formula) in the absence of other etiologies of kidney failure [11]. Diagnosis of coronary artery disease and heart failure was based on clinical or historical data or, examination by a trained cardiologist.

### 2.2 Statistical analysis

Statistical analysis was carried out using the Statistical Package for Social Science (SPSS 21.0) for Windows Software and Microsoft Excel 2019. The Kolmogorov-Smirnov test was used for testing for normal distribution of continuous variables, and the data were expressed as mean ± standard deviation. Categorical data are expressed as counts and percentages. For comparisons of categorical variables the Chi-square test was applied; continuous data was compared using unpaired Student t-tests or ANOVA.

One-way ANOVA was applied to determine the difference between the above defined groups. There was no outlier, as assessed by Box plot. Homogeneity of variance was violated, as assessed by the Levene test for equality of variance. Therefore, separate variance and Welch correction were used. A paired t-test was used to compare the means of the groups after treatment. A bivariate analysis between uncontrolled and controlled diabetes for all covariates and outcomes was performed (x^2^ test for categorical variables). Univariate and multivariate logistic regression was carried out to assess the association between uncontrolled hyperglycemia, microvascular complications, and other factors. For all analyses, a 2-sided value of p < 0.05 was considered statistically significant.

## 3 Results

A total of 12,069 patients was included in the analysis. The baseline characteristics of the patients are shown in Table 1. Mean age was 49.7 ± 11.3 years; male and female were almost equal in number. 2,891 (23.8%) subjects were aged less than 40 years. Mean duration of diabetes was 3.4 years, 9,566 (79.2%) patients had diabetes duration of less than 5 years, 9,306 (77.1%) were from urban areas, and 3,884 (32.2%) had a positive family history of diabetes.

**Figure 1.**
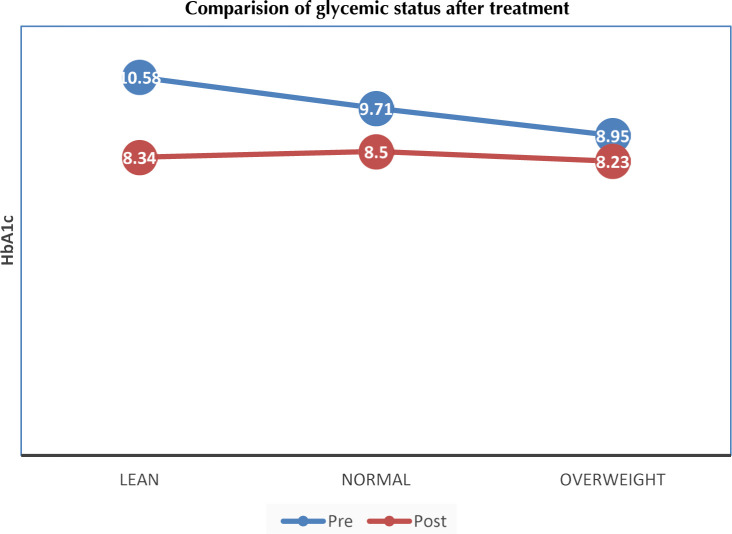
HbA1c at baseline and at follow-up in lean, normal weight, and overweight/obese patients.

Baseline fasting blood sugar, postprandial blood sugar, and HbA1c were 157.0 ± 68.0 mg/dl, 215.0 ± 90.0 mg/dl and 9.1 ± 2.3%, respectively. There was a significant decline in these values during the follow-up period (**Figure 1, Table 2**). Most of the patients (62.2%) were receiving two or three oral anti-diabetic drugs (**Table 1**). Metformin was the most commonly prescribed drug (94%) followed by sulfonylureas (55.7%). There was a significant positive correlation between HbA1c and age, presence of nephropathy and presence of retinopathy in bivariate analysis. HbA1c had a significant negative correlation with the number of oral anti-diabetic agents used.

**Table 1.** Baseline characteristics of study subjects

Parameters		Values
Age (yr.)		49.7 ± 11.3
Age category	< 40 yr	2876 (23.8)
	40-65 yr	8339 (69.1)
	>65 yr.	854 (7.1)
Gender	Male	6091 (50.5)
	Female	5978 (49.5)
Residence	Urban	9306 (77.1)
	Rural	2763 (28.9)
Family history of diabetes		3884 (32.2)
Diabetes duration (yr.)		3.4 ± 4.8
Duration Category	< 1 Year	5522 (45.5)
	1-5 Year	4044 (33.5)
	> 5 Year	2503 (20.7)
FBG (mg/dl)		157.2 ± 68.0
PPBG (mg/dl)		215 ± 90.2
HbA1c (%)		9.1 ± 2.3
Lipids (mg/dl)	TC	178.2 ± 47.9
	TG	184.6 ± 87.7
	HDL	45.3 ± 13.3
	LDL	95.3 ± 35.6
BMI (kg/m^2^)		27.2 ± 5.1
BMI category	Lean	327 (2.7)
	Normal-weight	1841 (15.3)
	Overweight	2310 (19.9)
	Obese	7591 (62.8)
Number of anti-diabetic drugs	One	3328 (27.6)
	Two	3455 (28.6)
	Three	4057 (33.6)
	Four	774 (6.4)
	Five	22 (0.5)
Kind of drugs	MFN	11384 (94.0%)
	SU	6717 (55.7)
	DPP-IV Inhibitors	5643 (46.8)
	SGLT-2	350 (2.9)
	Thiazolidinedione	74 (0.6)
	**α**-glucosidase inhibitor	1483 (12.30)
	Insulin	1070 (8.9)
Frequency of Insulin	Once daily	151 (14.1)
	Twice daily	749 (69.7)
	Thrice daily	39 (3.6)
	Four times daily	136 (12.6)
Neuropathy		1812(15)
Nephropathy		899 (7.4)
Retinopathy		1960 (16.2)
HTN		3108 (25.8)
CAD		1867 (15.5)

**Legend:** Data are mean ± SD or n (%) unless indicated otherwise. Abbreviations: BMI - body mass index; CAD - coronary artery disease; DPP-IV - dipeptidyl peptidase IV; FBG - fasting blood glucose; HbA1c - glycated hemoglobin; HDL - high-density lipoprotein; HTN - hypertension; LDL - low-density lipoprotein; MFN - metformin; PPBG - postprandial blood glucose; SGLT-2 - sodium-glucose cotransporter 2; SU - sulfonylureas; TC - total cholesterol; TG - triglycerides.

**Table 2.** Blood sugar fasting, postprandial and HbA1c values in lean, normal weight and overweight/obese patients at baseline and follow-up

-	p-value		Normal weight	p-value		Overweight	p-value		
	Baseline	Follow-up		Baseline	Follow-up		Baseline	Follow-up		
BS F	159.9 ± 78.3	142.6 ± 65.2	0.01	159.9 ± 73.9	144.1 ± 64.4	0.04	156.7 ± 66.8	128.1 ± 44.9	<0.001
BS PP	210.7 ± 77.2	175.1 ± 51.7	<0.001	223.1 ± 91.4	190.9 ± 98.6	<0.001	213.8 ± 81.5	169.4 ± 66.1	<0.001
HbA1c	10.6 ± 2.9	8.3 ± 1.6	<0.001	9.7 ± 2.5	8.5 ± 1.7	<0.001	8.6 ± 2.2	8.2 ± 1.6	<0.001

**Legend:** Data are mean ± SD or n (%) unless indicated otherwise. Abbreviations: BS (F): Blood sugar fasting; BS (PP): Blood sugar post prandial.

The American Diabetic Association guidelines were used to define the glycemic and non-glycemic targets [11], i.e.:

- Triglycerides: <150 mg/dl
- HDL cholesterol: >40 mg/dl for men and >50 mg/ dl for women
- LDL cholesterol: <70 mg/dl for patients with CAD and <100 mg/dl for patients without CAD
- Blood pressure: <140/90 mm of Hg

Out of total 12,069 patients 327 (2.7%) were lean, 1,841 (15.2%) had normal weight, and 9,906 (82.1%) were overweight/obese. The demographic, clinical and complication-related profiles of these patients are provided in Table 3. Lean patients were younger and had a shorter duration of diabetes compared with normal weight or overweight/obese patients. They also include more patients from rural areas and income of less than two lakh rupees (i.e. 200,000 rupees) per annum, which equals to US$ 2,700, approximately. The lean patients had a higher HbA1c at presentation and follow-up compared with overweight/obese patients.

**Table 3.** Characteristics of lean, normal weight and overweight/obese patients with type 2 diabetes mellitus

Parameters			Lean Mean ± SD	Normal Mean ± SD	Overweight Mean ± SD	ANOVA
Age			46.5 ± 14.1	50.7 ± 12.1	49.7 ± 10.9	< 0.001
Duration			2.7 ± 4.1	2.9 ± 4.7	3.5 ± 4.9	< 0.001
Body weight			43.9 ± 7.4	55.5 ± 7.6	73.0± 12.5	< 0.001
BMI			16.5 ± 1.5	21.1 ± 1.3	28.7 ± 4.3	< 0.001
Residence	Urban		168 (51.3)	1265 (68.7)	7873 (79.5)	< 0.001
	Rural		159 (48.7)	576 (31.3)	2028 (20.5)	
BSF (baseline)			159.9 ± 78.3	159.9 ± 73.9	156.7 ± 66.8	0.568
PP (baseline)			210.7 ± 77.2	223.1 ± 91.4	213.8 ± 81.5	0.116
HbA1c (baseline)			10.6 ± 2.9	9.7 ± 2.5	8.95 ± 2.2	< 0.001
Serum creatinine			1.08 ± .52	1.06 ± .37	1.07 ±.35	0.479
TC			181.3 ±51.9	177.1 ±48.2	178.2 ±47.7	0.723
TG			183.1 ± 79.2	177.1 ± 87.4	185.7 ±87.9	0.109
HDL			45.4 ± 13.3	44.3 ±11.7	44.4 ±11.8	0.703
LDL			98.50± 42.9	97.6 ± 34.9	95.6 ± 34.6	0.368
BSF (follow-up)			142.6 ± 65.2	144.2 ± 64.4	128.0 ± 44.9	0.005
BSPP (follow-up)			175.1 ± 51.7	190.9 ± 98.6	169.4 ± 66.1	0.157
HbA1c (follow-up)			8.3 ± 1.6	8.5 ± 1.7	8.2 ± 1.6	< 0.001
HbA1c reduction			2.72 ± 3.1	2.01 ± 2.6	1.30 ± 2.34	< 0.001
HbA1c < 7%			90 (27.5)	440 (23.9)	2767 (27.9)	0.002
Hypertension			47 (14.3)	371 (20.2)	2690 (27.2)	< 0.001
Nephropathy			20 (6.1)	121 (6.6)	758 (7.7)	0.17
Neuropathy			38 (11.6)	294 (15.9)	1480 ()14.9	0.11
Retinopathy			44 (13.5)	253 (13.7)	1648 (16.7)	0.003
CAD			37 (11.3)	231 (12.5)	1597 (16.1)	< 0.001
Duration		< 1 year	164 (50.1)	1005 (54.6)	4353 (43.9)	< 0.001
		1-5 years	112 (34.3)	525 (28.6)	3407 (34.4)	
		>5 years	51 (15.6)	311 (16.8)	2141 (21.7)	
Family history			78 (23.8)	505 (27.4)	3301 (33.3)	< 0.001
Metformin			299 (91.4)	1734 (94.1)	9315 (94.1)	0.35
Sulfonylureas			191 (58.4)	1008 (54.7)	5518 (55.7)	0.44
DPP-IV inhibitors			172 (52.6)	833 (45.4)	4638 (46.8)	0.04
α-glucosides inhibitor			35 (10.7)	202 (10.9)	1246 (12.6)	0.1
Insulin			31(9.4)	49 (2.5)	890 (8.9)	0.48

**Legend:** Data are mean ± SD or n (%) unless indicated otherwise. Abbreviations: BMI - body mass index; CAD - coronary artery disease; DPP-IV - dipeptidyl peptidase IV; FBG - fasting blood glucose; HbA1c - glycated hemoglobin; HDL - high-density lipoprotein; HTN - hypertension; LDL - low-density lipoprotein; MFN - metformin; PPBG - postprandial blood glucose; SGLT-2 - sodium-glucose cotransporter 2; SU - sulfonylureas; TC - total cholesterol; TG - triglycerides.

There was no significant difference in lipid parameters among the three groups. The prevalence of hypertension and family history of diabetes was less common in lean patients. Retinopathy was more common in lean patients, but there was no significant difference in the prevalence of nephropathy and neuropathy among the groups. Coronary artery disease was less common in the lean patients. There was no significant difference in the treatment prescribed among the three groups. Although the use of insulin was more common in lean patients, the difference was not statistically significant.

## 4 Discussion

The prevalence of diabetes is increasing in India. ICMR-INDIAB, a population-based study, has revealed that the prevalence of T2D is 7.3% in India [13]. In 2016, the prevalence was 5.2% in rural areas and 11.2% in the urban area [13]. The Southeast Asian T2D patient has a distinctive type of T2D that occurs in individuals with lower BMI, higher fat mass, higher insulin resistance, and higher inflammatory cytokine levels. The patients are 10-20 years younger than their western counterparts [14]. These factors are indicative of more severe diabetes and possibly of complications in Southeast Asian individuals [14-16]. Previous reports have revealed that a high percentage of Indian patients with diabetes is lean or of normal weight [17]. These subgroups of patients are not very well characterized.

We conducted this retrospective, cross-sectional study in 12,069 T2D patients living in northern Indian. The mean age of the patients was 49.7 ± 11.3 years; 2,876 (23.7%) patients were aged <40 years, 8,389 (69.1%) were between 40-65 years, and 854 (7.1%) were >65 years of age. This finding is similar to previous studies conducted in India [18-20], but it is different from those obtained in western populations, where elderly patients form a large chunk of the diabetic population [21]. This also shows that diabetes occurs at a younger age in Indians compared to other populations. The number of male participants was 6,091 (50.5%), which is similar to previous studies [22]; 9,901 (82.0%) patients were either overweight or obese, which is also similar to recent observations in India [23].

327 (2.7%) of the patients were lean, 1,841 (15.3%) had normal body weight, and 9,901 (82%) were overweight or obese. In a study from southern India, Mohan *et al*. reported an incidence of 3.5%, 63.5%, and 32.9% for lean, normal weight and obese T2D individuals, respectively [5]. But they used different criteria for the definition of obesity, namely BMI >27 kg/m^2^ for men and >25 kg/m^2^ for women. In our study, 82% of the patients were overweight or obese. This difference between the studies may be due to the different cut-off values used to define obesity. Another reason could be the increased prevalence of obesity in India, as Mohan and coworkers conducted their study more than twenty years ago [5].

In our study, lean patients were younger than patients in the other two groups. This finding is in contrast to the previous two studies from India [5, 24]. However, patients in both these studies had a longer duration of diabetes than in our study. Findings similar to our study have been reported from countries other than India [25]. This suggests a strong genetic predisposition in these lean T2D patients. The level of fasting blood glucose, postprandial blood glucose, and HbA1c was significantly higher in lean and normal weight than in overweight/obese patients at presentation. This finding is similar to previous findings and indicates a more aggressive disease [5]. There was a significant decline in the levels of fasting blood glucose, postprandial blood glucose, and HbA1c during follow-up in all three groups of patients (**Table 2**). But during follow-up, lean and normal-weight patients had significantly higher fasting blood glucose and HbA1c levels than overweight/obese patients. A higher number of overweight and obese patients achieved HbA1c levels of less than 7% and a lower number of these patients had an HbA1c of more than 9% compared with lean and normal-weight patients. This indicates that glycemic control was better in obese T2D than in lean and normal-weight T2D patients. These findings are similar to previous results obtained in India [5].

The lean group had a significantly higher number of patients from rural areas and lower income groups than the two other groups (**Table 3**). This indicates that the poverty leading to malnutrition in early life may predispose for the development of diabetes. Slim healthy Caucasians with a history of low birth weight have been shown to develop T2D pathophysiological abnormalities, such as decreased insulin secretion, decreased uptake of glucose by muscle, decline in insulin-mediated glycolysis, and augmented fat deposition, earlier in life than others [26, 27]. The Dutch Famine study has also shown that malnutrition in early life is associated with increased risk of diabetes [28]. Similar findings have been reported in different Asian populations [25].

The overweight/obese group had a stronger family history of diabetes. It has been demonstrated that obese individuals with a family history of diabetes have a higher rate of diabetes than those with negative family history [29]. The incidence of hypertension and coronary artery disease was significantly increased in the overweight/obese group. Similar findings have been reported by Mohan *et al*. [5]. There was no significant difference in the pattern of oral antidiabetic drugs and insulin prescription between the three groups. This finding is different from earlier findings from India, which showed a higher use of insulin in the lean group [7, 24]. The difference may be explained by the fact that the lean group in the above study had mean diabetes duration of 9.2 years, while in our study group it was only 2.7 years, and 50% of patients in the lean group had a duration of <1 year. With longer duration of follow-up they might have increased insulin requirement.

There was a significant decline in fasting blood glucose, postprandial blood glucose, and HbA1c in the three groups during follow-up. Although the decrease in HbA1c was more distinct in the lean group, there was a significantly better control of hyperglycemia in the overweight/obese group (**Figure 1**). This indicates that we need intense treatment in the lean and normal-weight group to improve glycemic control.

The major factor leading to hyperglycemia in lean T2D patients is impaired secretion of insulin from pancreatic β-cells [25]. This could be secondary to the phenomenon of small β-cells found in the autopsies of lean individuals [25]. The findings from our study indicate that, at least in the initial stage, the treatment regimen was similar in all the groups. The inverse correlation between the number of oral anti-diabetic drugs and HbA1c indicates that early aggressive treatment was associated with better glycemic control.

The use of metformin was relatively equal in all the groups: >90% of patients (91% in the lean group and 94% each in the normal-weight and overweight/ obese group). This indicates that metformin was well tolerated in the lean T2D group. Previous studies have shown that metformin is effective in controlling hyperglycemia in both lean and obese T2D patients [30]. There was no significant difference in the levels of total cholesterol, triglycerides, LDL cholesterol and HDL cholesterol between the groups, which is not in agreement with findings of previous studies [5, 24]. This may be due to the different criteria used to define obesity and the age of the subjects in these studies, as the latter were older than those in our study.

There was no significant difference in the rate of diabetic neuropathy and nephropathy between the groups. Previous reports from India have reported a high incidence of microvascular complications in lean T2D individuals [5, 24]. This discordance of observations may be due to younger age and lesser duration of diabetes in lean T2D subjects in our study. The incidence of retinopathy was higher in the obese group, which may be secondary to the higher duration of diabetes in this subgroup.

The strength of our study is that it was a multicenter study, with subjects from both government and private setups. This lessens the chances of an inclusion bias. Also, a large number of patients were included in the analysis, and comprehensive data regarding demography, clinical variables, biochemical parameters and both microvascular and macrovascular complications were obtained. The limitations are that it was a hospital-based retrospective study, which includes the risk of bias, and a cause and effect relationship cannot be established.

## 5 Conclusions

T2D is a heterogeneous clinical phenomenon. We analyzed the clinical features and response to standard treatment in a large cohort of lean, normal-weight, and overweight/obese T2D patients from northern India. The overall achievements of various glycemic and nonglycemic targets in T2D patients were suboptimal. Our study revealed that lean and normal-weight individuals have more severe hyperglycemia and relatively poor response to treatment, which indicates that diabetes in such individuals may represent a more aggressive form of the disease. Patients in all three subgroups were relatively young, which suggests the need for aggressive treatment of diabetes. More prospective studies are needed for further delineation of the natural history and appropriate treatment in the lean T2D patient.
